# Genotype imputation performance in Nellore cattle across different SNP panels and software tools

**DOI:** 10.1007/s11250-026-04914-0

**Published:** 2026-03-12

**Authors:** G. Campos, H. A. Mulim, H. Ventura, N. Souza, F. Cardoso, H. R. Oliveira

**Affiliations:** 1 Department of Animal Sciences, Purdue University, West Lafayette, IN 47907 USA; 2 Brazilian Association of Zebu Breeders, Uberaba, MG 38022-330 Brazil; 3 Embrapa Pecuária Sul, Bagé, RS 96401-970 Brazil

**Keywords:** *Bos taurus indicus*, Computational efficiency, Imputation accuracy, Nelore

## Abstract

Most commercial genotyping panels were designed for *Bos taurus taurus* breeds, which may reduce their informativeness in *Bos taurus indicus* populations like Nellore due to differences in linkage disequilibrium and minor allele frequencies. Genotype imputation is a cost-effective method to increase marker density by predicting missing genotypes in low- and medium-density (LD and MD) SNP panels using high-density (HD) reference data. While extensively studied in *Bos taurus taurus* breeds, limited data exist for Nellore cattle. This study evaluated the imputation performance of four widely used software tools—FImpute, Beagle, Minimac, and Findhap—using a large dataset of over 305,000 Nellore animals genotyped by the Brazilian Association of Zebu Breeders (ABCZ). Imputation was performed in two steps: from LD/MD panels to a customized 120k panel, then to a 777k HD panel. Accuracy was measured using Pearson correlation (Corr), the percentage of correctly imputed genotypes (PERC), and computational performance. All software achieved high accuracy (Corr: 0.82–0.98; PERC: 89.35%–99.26%). FImpute consistently showed the best performance, with the highest Corr (0.983), the fewest poorly imputed individuals, and strong results across all panel densities. While pedigree information in FImpute offered some benefits, its overall improvement compared to population-based methods was limited in this study. Imputation accuracy improved with panel density and was stable across most MAF ranges, though rare allele (MAF ≤ 0.03) imputation remained challenging. These findings confirm that accurate genotype imputation in Nellore cattle is feasible using existing commercial SNP panels.

**Supplementary Information:**

The online version contains supplementary material available at https://doi.org/10.1007/s11250-026-04914-0.

## Introduction

Genomic selection has revolutionized livestock breeding by enabling earlier and more accurate identification of superior animals (Hayes et al. 2009; Meuwissen et al. 2016). This is particularly important in tropical regions such as Brazil, where Nellore cattle (a *Bos taurus indicus* breed) dominate beef production and play a key role in ensuring food security and the sustainability of the livestock industry (FAO 2023). Efficient use of genomic tools in these populations can significantly improve selection for economically important traits such as growth, carcass-related traits (Silva Neto et al. 2023), and fertility (Alves et al. 2021).

The rapid advancement of sequencing and genomic technologies has led to the development of various SNP panels with different densities across multiple platforms (Ventura et al. 2019). However, most commercially available genotyping platforms were developed using *Bos taurus taurus* (Taurine) breeds as reference populations (Ventura et al. 2019). Consequently, these panels may be less effective in *Bos taurus indicus* (Zebu) cattle due to differences in linkage disequilibrium patterns and minor allele frequencies (MAF) between the two subspecies (Pérez O’Brien et al. 2014). The efficacy of SNP panels for genomic studies depends largely on the linkage disequilibrium between markers and quantitative trait loci (QTL), which is influenced by the number and informativeness of SNP markers. For example, if many SNPs included in a panel designed for Taurine cattle are fixed in Zebu populations, they are likely to be filtered out during quality control, resulting in fewer usable markers and reduced power for genomic prediction and association studies (Ventura et al. 2019).

One strategy to address this limitation is genotype imputation, which uses information from a high-density reference population to infer missing genotypes in animals genotyped with lower-density SNP panels (Hayes et al. 2012). While several studies have evaluated the performance of imputation software in *Bos taurus taurus* beef breeds (e.g., Piccoli et al. 2014; Campos et al. 2022), there is limited information on their effectiveness in Nellore cattle genotyped with the diverse number of SNP panels currently available for commercial use (Carvalheiro et al. 2014; Espigolan et al. 2013; Bernardes et al. 2019). Recent efforts have mainly evaluated genotype imputation in Nellore cattle using whole-genome sequence (WGS) data. For instance, Fernandes Júnior et al. (2021) demonstrated the potential of sequence-level imputation in key ancestral animals. While that study focused on WGS imputation, the present work complements those findings by evaluating imputation accuracy across commercial SNP chip densities widely used in routine breeding programs. Therefore, the objective of this study was to evaluate imputation accuracy in Nellore cattle raised in Brazil using the official dataset from the Brazilian Association of Zebu Breeders (ABCZ), by comparing 14 commercial SNP panels and four different imputation software programs.

## Materials and methods

### Pedigree and genotypic data

The ABCZ provided pedigree information for about 14 million Nellore animals raised in Brazil. Out of these, 309,640 animals were genotyped using one of 14 commercially available SNP panels, which were categorized into low-density (LD), medium-density (MD), and high-density (HD) groups. The LD group included the 14k, 22k, 26k, 27k, 29k, 30k, 35k1, and 35k2 SNP panels. The MD group included the 50k1, 50k2, 70k1, and 70k2 SNP panels; and the HD group included only the panel with 777k SNPs. Details about the number of SNPs and genotyped animals per SNP panel are shown in the Supplementary Material (Table S1). A total of 618,596 animals were either genotyped and/or related to the genotyped animals and were available to be included in the analyses. All genotyped animals were represented in the pedigree file, although some individuals had incomplete parent information. A summary of the pedigree for the genotyped animals is shown in Table 1.

**Table 1:** Summary of the pedigree structure and genotyped animals

Class	Numbers
*Pedigree*	
Total individuals	618,596
Sires	29,258
Dams	327,290
Individuals with only known sire	55,432
Individuals with only known dam	50,823
Individuals with known sire and dam	554,183
Individuals with unknown sire and dam	41,842
*Genotypes*	
Total genotyped individuals	305,184
Genotyped males	128,144
Genotyped females	174,221
Genotyped sires	3,344
Genotyped dams	43,446
Individuals with only known sire	8,364
Individuals with only known dam	13,773
Individuals with known sire and dam	289,938
Individuals with unknown sire and dam	6,894

### Genotypic quality control

**Fig. 1:**
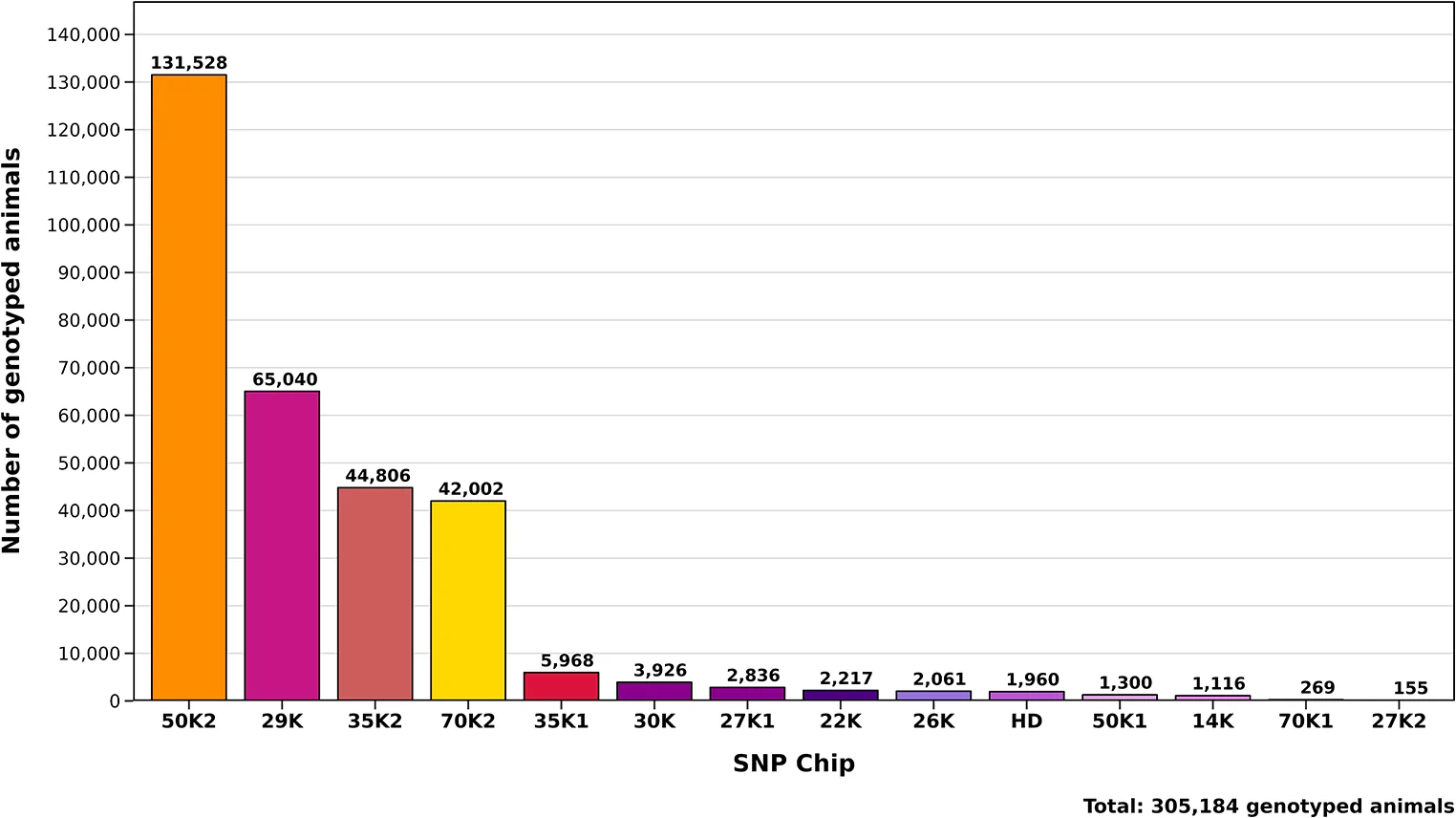
Final number of genotyped animals per SNP panel

Quality control of genotypes was performed independently for each SNP panel using the QCf90 software (Masuda et al. 2019). All SNP marker positions were updated to the ARS-UCD1.2 bovine reference genome assembly (Rosen et al. 2020). The SNP map coordinates were updated using the rtracklayer R package (Lawrence et al. 2009), which performs LiftOver through UCSC chain files. Following the standard ABCZ genomic evaluation pipeline, SNPs with call rate < 0.90, MAF < 0.01, or strong deviation from Hardy–Weinberg equilibrium (HWE < 0.15) were removed. These filters are necessary because many SNPs originally designed for *Bos taurus taurus* breeds are monomorphic or nearly fixed in Nellore cattle, and may introduce noise or technical artifacts when included in imputation analyses. Despite this filtering, the HD panel retained a substantial proportion of rare variants (16.5% with MAF ≤ 0.03), allowing us to evaluate rare-allele imputation performance. The number of overlapping SNPs between SNP panels after quality control is shown in the Supplementary Material (Table S2). The final number of genotyped animals per SNP panel is shown in Fig. 1. After all quality control steps, 305,184 genotyped animals imputed to a density of 617,225 SNPs were retained for subsequent imputation analyses.

### Imputation

In this study, different imputation tools and panels were evaluated to determine the most appropriate ones for this Nellore cattle population. A total of four different software, i.e., Fimpute v3 (Sargolzaei et al. 2014), Findhap (VanRaden et al. 2015), Minimac4 (Das et al. 2016), and Beagle v.5.4 (Browning et al. 2018) were tested in this study. For Minimac4, the reference and validation sets were phased separately using Eagle v2 (Ref). In addition, to be more efficient, Minimac4 requires reference panels in M3VCF format, which were obtained using Minimac3 (https://genome.sph.umich.edu/wiki/Minimac4). In contrast, for imputations performed with Beagle, the software itself was used for both phasing and imputing reference and validation sets. As the Fimpute software allows the use of the deterministic algorithm based either on family or population information to impute the missing genotypes, both methods were contrasted using FImpute (i.e., with and without pedigree information).

The imputation was performed in two steps. First, all LD and MD genotypes were imputed to a custom SNP panel containing 120,615 unique SNPs. This panel was created by merging all unique SNPs present in the post-QC of two versions of the 50k and two versions of the 70k panels and removing duplicates after harmonizing genomic positions. A full summary of overlapping SNPs between this custom panel and each LD/MD panel, as well as the HD panel, is now provided in Supplementary Table S3. After this step, the 120k SNP panel was imputed to the HD panel. The imputation for the whole dataset (i.e., 305,184 genotyped animals after quality control) was only performed using the software with the optimal overall performance for this population, which was assessed by allele/genotype imputation accuracy and computational efficiency.

#### Overall imputation performance

To evaluate the overall imputation performance of each software, animals genotyped with the HD panel (*N* = 1,960) were randomly split into reference (*N* = 1,567 animals) and validation (*N* = 393 animals) sets. In the validation set, HD genotypes were masked to mimic the thirteen LD and MD SNP panels under evaluation in this study (i.e., each imputation analysis was repeated thirteen times, one for each LD/MD SNP panel). Thereafter, the ability to impute the correct allele/genotype was assessed using the accuracy of imputation, calculated based on the Pearson correlation (Corr) coefficient estimated between true and imputed genotypes at a specific locus, and the percentage of correctly imputed genotypes (PERC). Genotypes were coded as 0, 1, or 2; corresponding to the homozygous reference allele, heterozygous genotype, or homozygous alternative allele, respectively. The Corr equation can be described as follows:

$$\:{\mathrm{C}\mathrm{o}\mathrm{r}\mathrm{r}}_{\mathrm{i}}=\frac{\mathrm{C}\mathrm{o}\mathrm{v}({\mathrm{X}}_{\mathrm{i}},{\mathrm{Y}}_{\mathrm{i}})}{\sqrt{\mathrm{V}\mathrm{a}\mathrm{r}\left({\mathrm{X}}_{\mathrm{i}}\right)\mathrm{V}\mathrm{a}\mathrm{r}\left({\mathrm{Y}}_{\mathrm{i}}\right)}},\:$$

where Corr_i_ is the imputation accuracy; X_i_ is the vector of imputed genotypes, and Y_i_ is the vector of the true genotypes for a specific locus i. The PERC was calculated as follows:

$$\:\mathrm{P}\mathrm{E}\mathrm{R}\mathrm{C}\left(\mathrm{\%}\right)=\frac{{\mathrm{N}}_{\mathrm{c}\mathrm{o}\mathrm{r}\mathrm{r}\mathrm{e}\mathrm{c}\mathrm{t}\mathrm{l}\mathrm{y}\:\mathrm{i}\mathrm{m}\mathrm{p}\mathrm{u}\mathrm{t}\mathrm{e}\mathrm{d}}}{{\mathrm{N}}_{\mathrm{t}\mathrm{o}\mathrm{t}\mathrm{a}\mathrm{l}\:\mathrm{i}\mathrm{m}\mathrm{p}\mathrm{u}\mathrm{t}\mathrm{e}\mathrm{d}}}\times100,$$

where $\:{\mathrm{N}}_{\mathrm{c}\mathrm{o}\mathrm{r}\mathrm{r}\mathrm{e}\mathrm{c}\mathrm{t}\mathrm{l}\mathrm{y}\:\mathrm{i}\mathrm{m}\mathrm{p}\mathrm{u}\mathrm{t}\mathrm{e}\mathrm{d}}$ is the number of correctly imputed alleles and $\:{\mathrm{N}}_{\mathrm{t}\mathrm{o}\mathrm{t}\mathrm{a}\mathrm{l}\:\mathrm{i}\mathrm{m}\mathrm{p}\mathrm{u}\mathrm{t}\mathrm{e}\mathrm{d}}$ is the total number of alleles imputed. Both Corr and PERC were calculated based only on samples and SNPs that had imputed genotypes. Additionally, we analyzed the impact of MAF for each scenario to determine the performance of different software on imputing rare alleles (MAF ≤ 0.03).

The computational efficiency of each program was assessed based on the time and memory usage (in gigabytes) for imputation. Time and memory usage were recorded using the module load monitor, which is built with the resource-monitor package in Python. All analyses were performed using the Negishi server at Purdue University. Negishi nodes run on Rocky Linux 8 with 128 processor cores, 256 GB of memory, and 100 Gbps Infiniband interconnects. More information about Negishi is available at the Purdue Negishi website (www.rcac.purdue.edu/compute/negishi). To ensure a comparable assessment of processing-time efficiency, each software was executed by parallelizing the 29 chromosomes across 58 processors, following an approach similar to that described by Fernandes Júnior et al. (2021). All software used in this study supports multithreaded processing with the following options: FImpute (--njob), Beagle (--nthreads), Minimac4 (--cpus), and Findhap (threads, command-line argument).

## Results

### Imputation accuracy and percentage of correctly imputed genotypes by SNP panel and software

**Fig. 2:**
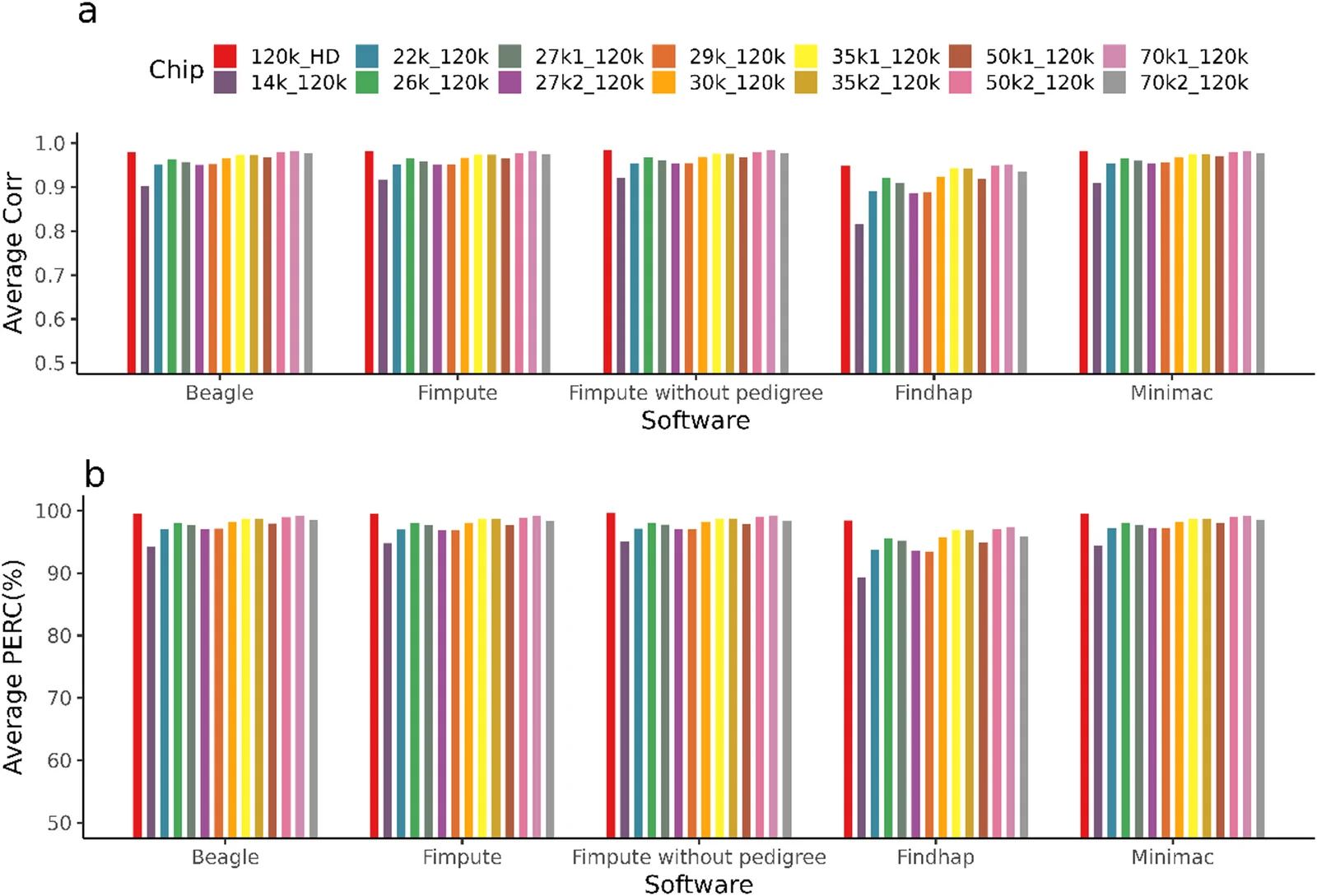
Average accuracy of SNP imputation for different scenarios, software, and imputation measurements. Average Corr: Average Pearson correlation coefficient estimated between true and imputed genotypes at a specific locus. Average PERC (%): Average percentage of correctly imputed genotypes

The Corr and PERC were generally high across all scenarios (Fig. 2). Corr values ranged from 0.82 (14k panel, Findhap) to 0.98 (70k1 panel, FImpute), and PERC ranged from 89.35% to 99.26%. Comparison of Corr and PERC values for FImpute, with and without pedigree information, indicated no significant increase in accuracy when pedigree information was used for imputation. As Corr and PERC yielded similar results (in terms of ranks of programs/SNP panels), subsequent results will focus solely on Corr.

The average Corr was slightly higher for imputation performed from the MD (0.023 units higher) to the 120k SNP panel, compared to the imputation from LD SNP panels to the 120k panel. Consequently, imputation accuracy increased as the number of SNPs to be imputed decreased. The Corr values estimated for Fimpute, Beagle, and Minimac were similar, ranging from 0.981 to 0.984. Among all software, Findhap had the lowest Corr (0.948). Despite the number of SNPs to be imputed from the 120k to the HD panel (i.e., 496,610 SNPs), this scenario exhibited the highest accuracy compared to the others. The lowest Corr was obtained with the 14k SNP panel using Findhap, and the highest Corr was achieved using the 70k1 SNP panel and the Fimpute software.

**Fig. 3:**
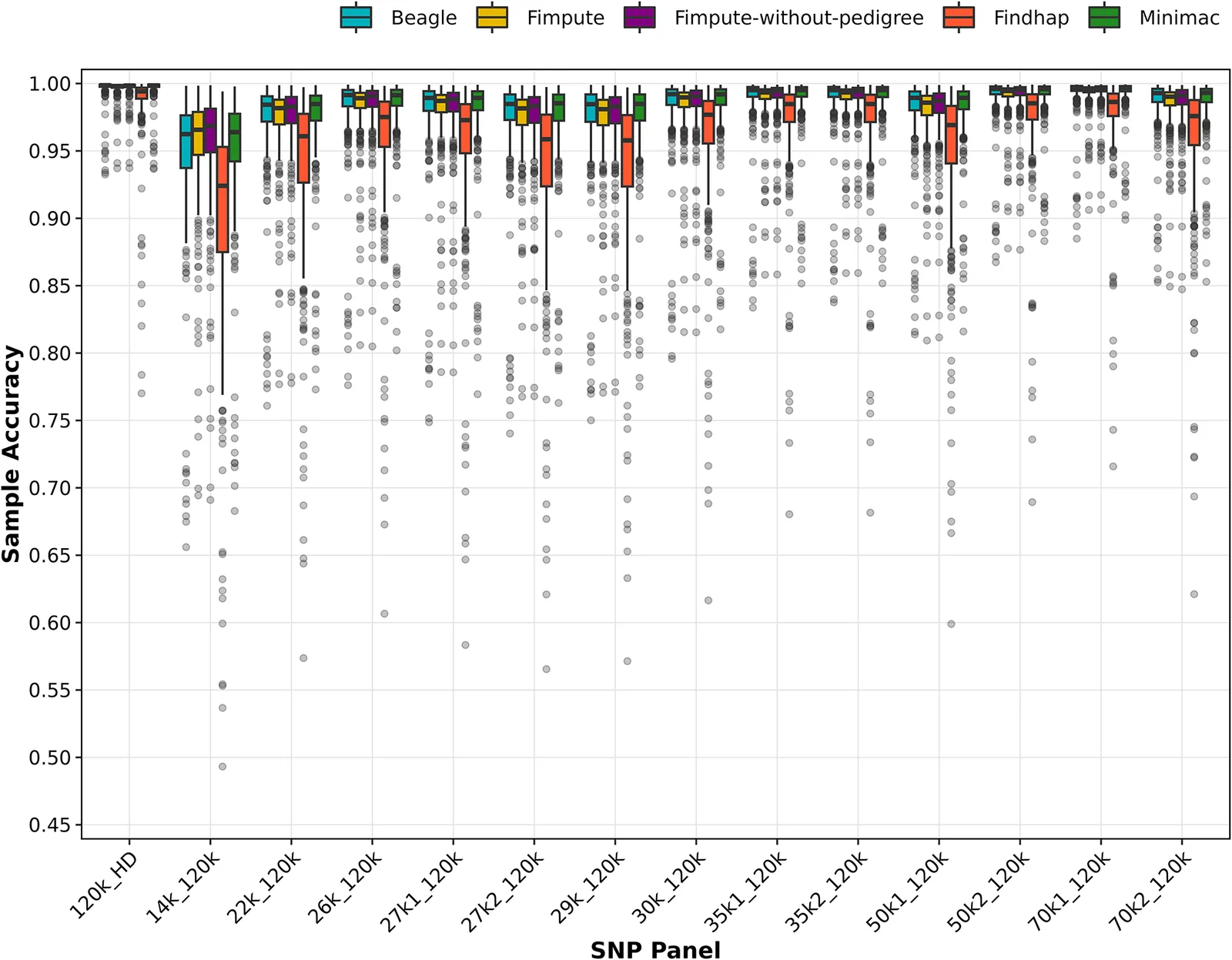
Distribution of individual imputation accuracies (Pearson correlation coefficient) for Nellore cattle when imputing from various low-density and medium-density SNP panels to a 120k SNP panel, compared across different imputation software (FImpute, Beagle, Minimac, and Findhap)

The distribution of individual accuracies for all SNP panels and software is shown in Fig. 3. For LD panels, the average Corr ranged from to 0.950 (Findhap) to 0.979 (Fimpute). However, on average, the 14k panel had the lowest Corr values among all SNP chips. The Findhap software had the highest number of individuals with accuracy lower than 0.80 (*N* = 112 animals), while the FImpute software had the lowest (*N* = 10 animals).

### Impact of minor allele frequencies on imputation

**Fig. 4:**
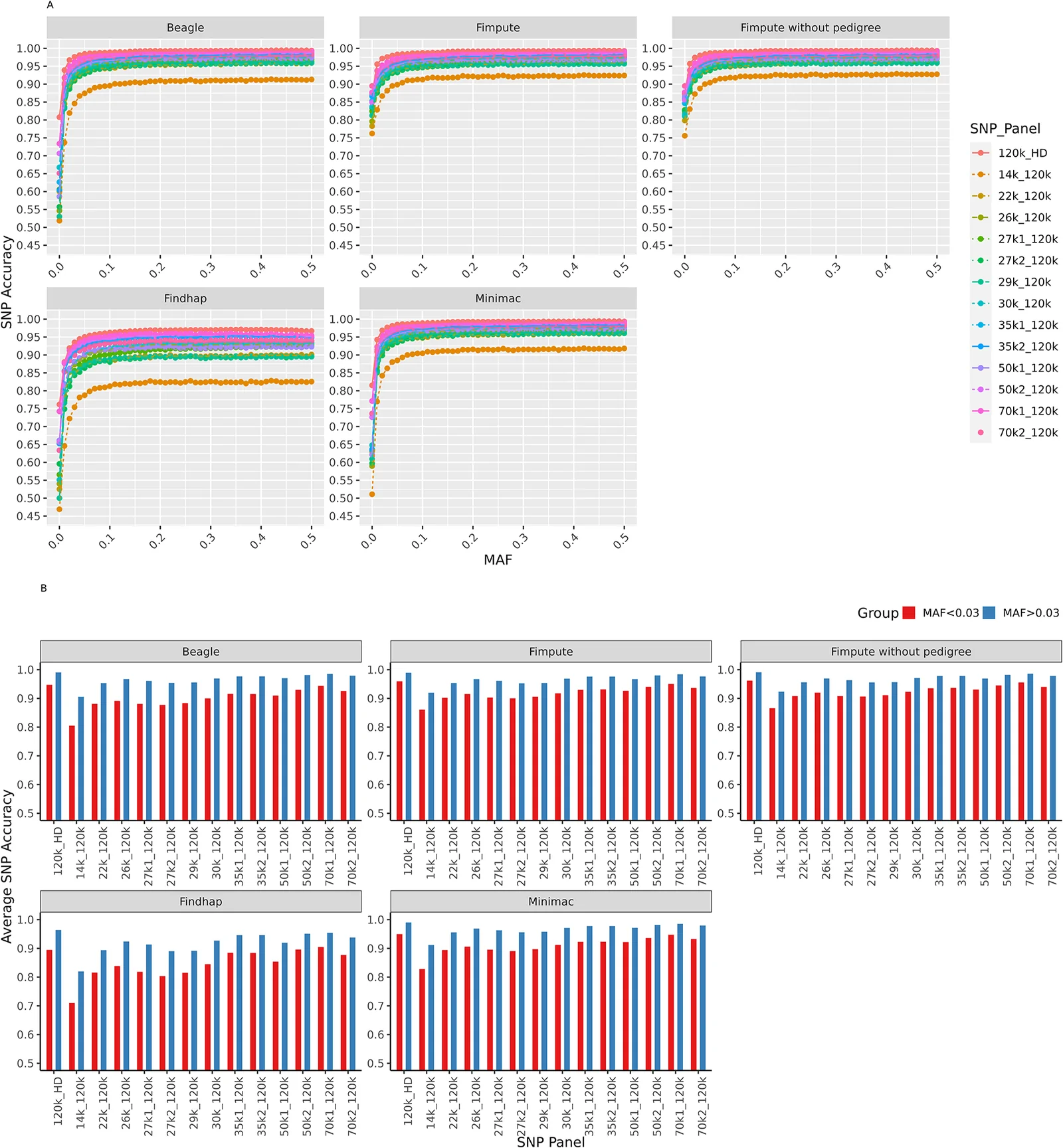
(**A**) SNP imputation accuracies by minor allele frequency (MAF) for each software and panel; and (**B**) average SNP accuracy for rare (MAF < 0.03) and common (MAF > 0.03) variants, using different software and SNP panels

The imputation of rare alleles is a crucial factor that affects the prediction of missing genotypes, regardless of the population. In fact, 16.5% of the total number of SNPs in our study were rare variants (MAF ≤ 0.03). A visual representation of the MAF distribution for the HD panel is included in the Supplementary Material (Figure S1). Figure 4A shows the relationship between the MAF and imputation accuracy for various SNP panels and software. The trend between Corr and MAF was found to be similar for all SNP panels. As expected, the increase in MAF resulted in an increase in imputation accuracy, suggesting that it is more difficult to impute rare alleles compared to common ones.

FImpute has proven to be more effective in imputing rare alleles compared to the other software (Fig. 4A). Considering only the imputation from LD/MD to the 120k SNP panel, FImpute without pedigree, FImpute with pedigree, Minimac, Beagle, and Findhap had an average Corr of 0.921, 0.916, 0.908, 0.896, and 0.842, respectively. However, when considering the imputation from 120k to the HD SNP panel, the average accuracy for rare alleles was higher for all the software, ranging from 0.894 (Findhap) to 0.961 (Fimpute), suggesting that the 120k panel has enough information for imputing SNPs with low MAF. Regarding the SNP panels, the 14k had the lowest accuracies for both rare and common alleles, which was expected because this is the lowest density SNP panel included in our study. In this context, all tested software had imputation accuracies higher than 0.80 for the 14k and MAF ≤ 0.03, except Findhap (the average Corr value for Findhap was 0.701; as shown in Fig. 4B).

### Computational performance

**Fig. 5:**
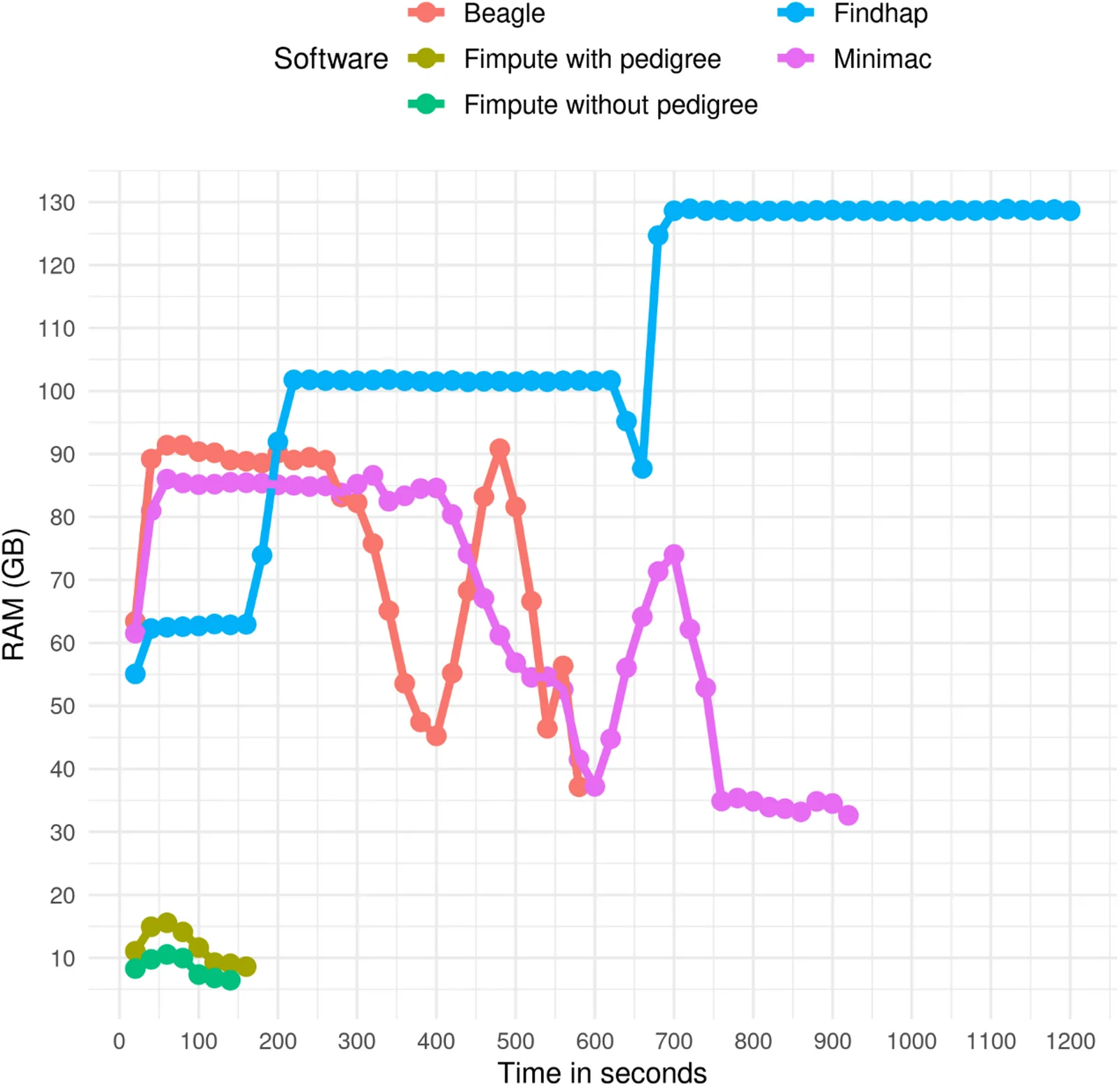
CPU time (in seconds) and memory usage for different software to impute from 120k SNPs to high-density panel

We tested different imputation software by comparing the time and RAM usage required to complete the imputation. To perform the comparison, we selected the scenario with the most SNPs to be imputed (120k to HD). We parallelized the imputation of the 29 bovine chromosomes using 58 processors (two processors per chromosome). Figure 5 shows the CPU time and memory usage for the different software. Fimpute with pedigree took 2 min and 4 s to complete the imputation, while Fimpute without pedigree took 1 min and 45 s. On the other hand, Beagle, Minimac, and Findhap took 11 min and 51 s, 16 min and 17 s, and 23 min and 4 s, respectively. We also compared the memory usage of different software by measuring the amount of RAM allocated in gigabytes. Figure 5 shows that Fimpute used an average of 12.57 gigabytes with pedigree and 8.87 gigabytes without pedigree. However, Beagle, Minimac, and Findhap used 76.30, 65.81, and 110.13 gigabytes, respectively. Therefore, Fimpute proved to be faster and uses less memory than the other imputation software tested in this study.

## Discussion

Few studies have evaluated the efficacy of imputation using different software in indicine cattle (e.g., Carvalheiro et al. 2014; Hermisdorff et al. 2020). However, marker density significantly impacts imputation accuracy, and the differences in linkage disequilibrium between indicine and taurine breeds (Pérez O’Brien et al. 2014) might result in different performance of imputation for both species. As a result, investigating the performance of different imputation software for Nellore cattle using different SNP panels is paramount.

Apart from the 14k panel, imputation accuracy remained high for all other LD or MD panels up to 120k (Fig. 2). Previous studies, such as those by Pausch et al. (2012) and Badke et al. (2012), have shown that imputation accuracy improves as the density of the LD panel increases. Carvalheiro et al. (2014) assessed the imputation accuracy of various commercial and customized LD panels (ranging from 7k to 75k) to an HD panel in Nellore cattle, and they found similar accuracies compared to our study, despite imputing directly to 777k SNPs. They suggested that genotyping animals with a SNP panel containing less than 15k SNPs is not recommended for Nellore cattle. Based on our findings, we also recommend that the 14k panel should not be used for Nellore cattle. An alternative approach could involve imputing the 14k panel to an MD panel and then to the customized panel (120k) to enhance the accuracy of this SNP panel. However, we did not directly test the one-step imputation on the HD panel.

Despite the approach, the software used also impacts the accuracy of the imputation. In our study, the Fimpute software showed the best performance, followed by Beagle, Minimac, and Findhap (Figs. 3 and 4). For Nellore cattle, Carvalheiro et al. (2014) found that Fimpute outperformed Beagle for all LD chips and validation sets when imputing to an HD panel. In a composite beef cattle population, Chud et al. (2015) reported a 5% increase in imputation accuracy using the Fimpute algorithm over Beagle when imputing from the 9k to the HD SNP panel. Ma et al. (2013) compared five imputation software (i.e., IMPUTE2, Beagle, Findhap, AlphaImpute, and Fimpute) in Swedish and Finnish Red Dairy Cattle. They found that IMPUTE2 and Beagle resulted in higher accuracies and were more robust under various conditions when imputing from 3 K to MD (54k). However, the authors highlighted that when imputing from MD to HD, the accuracy of imputation using Fimpute was similar to that of Beagle and IMPUTE2 and higher than that of the other methods.

Regardless, Fimpute was the most computationally efficient in terms of time and memory compared to the other software we tested (Fig. 5). Other studies using different SNP panels (Ma et al. 2013; Piccoli et al. 2014; Sargolzaei et al. 2014) and sequence data (Fernandes Júnior et al. 2021) reported that Fimpute performed significantly better. Despite the software using population methods (e.g., Beagle and Minimac) showing similar accuracies, their main drawbacks were long running times and high memory usage compared to Fimpute. In our study, Findhap demonstrated higher time and memory usage requirements than all other software.

Imputing alleles with very low minor allele frequency remains one of the most challenging aspects of genotype inference. Rare variants frequently include functional or deleterious mutations that can substantially influence economically important traits. Recent studies in Nellore cattle have identified rare lethal haplotypes using imputed high-density genotypes (Schmidt et al. 2023; Rodrigues et al. 2025), demonstrating the importance of high-quality imputation for low-frequency alleles. Poorly imputed rare variants may remain undetected or bias genomic evaluations. In our study, FImpute showed the most stable performance across MAF classes, underscoring its suitability for analyses where precise recovery of rare alleles is critical.

It is crucial to accurately impute these rare alleles for association studies because they can account for a large proportion of variation not explained by common alleles(Cirulli and Goldstein 2010) and consequently, many causal mutations for complex traits may be present in this group of alleles (Manolio et al. 2009). We observed that different imputation software displayed varying accuracy of imputation according to changes in MAF (Fig. 4). In our study, Fimpute exhibited the least fluctuation in accuracy with changes in MAF compared to other software, indicating its superior efficiency in imputing missing genotypes with low MAF. This is attributed to Fimpute’s ability to robustly identify long haplotype matches where rare alleles are likely situated (Hirschhorn and Daly 2005). Our findings support the results of other studies. For instance, Fimpute showed better performance for rare variants compared to different software, such as Beagle, Findhap, Impute2, and AlphaImpute, when imputing from 54k to HD (Ma et al. 2013). In Nellore cattle, Fernandes Júnior et al. (2021) also observed higher accuracy in imputing rare alleles from HD to sequence data with Fimpute compared to Minimac. In most studies, methods based on pedigree are more effective in identifying rare alleles than population-based methods. This is because pedigree-based methods like Fimpute take into account the transmission of identical-by-descent (IBD) genomic segments through the pedigree structure (Phocas 2022), allowing for the reliable identification of rare alleles within these segments. Although Findhap uses a combination of family and population-based algorithms to impute missing genotypes, the software showed the worst performance in imputing rare alleles, possibly due to its lower efficiency in finding long haplotype segments compared to Fimpute.

Regarding the use of pedigree information for imputation, Fimpute showed no significant difference whether the pedigree was included or not. This is likely related to the high quality of pedigree (the proportion of unknown sires was 4% considering only genotyped animals and 8% considering all individuals), as well as the large number of animals genotyped over the years. Consequently, Fimpute (with or without pedigree) is recommended as the most suitable software for imputing several SNP panels in this Brazilian Nellore cattle population.

## Supplementary Information

Below is the link to the electronic supplementary material.


Supplementary Material 1


## Data Availability

The datasets analyzed in this study were provided by the Brazilian Association of Zebu Breeders (ABCZ) and are not publicly available due to data ownership restrictions and confidentiality agreements. However, the datasets may be available from Dr. Henrique Ventura (henrique@abcz.org.br) upon reasonable request.
