# Supplementary material for: Genotype imputation performance in Nellore cattle across different SNP panels and software tools
Source: Trop Anim Health Prod. 2026 Mar 12;58(2):172. doi: 10.1007/s11250-026-04914-0 (PMC12982288; doi:10.1007/s11250-026-04914-0)
Supplement: Supplementary file 1 — Supplementary Material 1 [file 11250_2026_4914_MOESM1_ESM.docx]

**Genotype Imputation Performance in Nellore Cattle Across Different SNP Panels and Software Tools**

G. Campos; H. A. Mulim; H. Ventura; N. Souza; F. Cardoso; H. R. Oliveira

**Supplementary Material**

**Table S1:**  **Number of SNPs and animals before quality control for each SNP panel.**

| **SNP Panel** | **SNPs** | **Samples** |
| --- | --- | --- |
| **14k** | 14,409 | 1,123 |
| **22k** | 18,285 | 2,219 |
| **26k** | 24,326 | 2,123 |
| **27k_1** | 25,879 | 2,839 |
| **27k_2** | 27,214 | 155 |
| **29k** | 26,551 | 65,577 |
| **30k** | 27,952 | 4,110 |
| **35k_1** | 33,467 | 46,594 |
| **35k_2** | 33,426 | 6,114 |
| **50k_1** | 51,924 | 1,313 |
| **50k_2** | 51,546 | 42,381 |
| **70k_1** | 69,610 | 276 |
| **70k_2** | 65,354 | 42,381 |
| **HD** | 733,676 | 2,055 |

**Table S2:** **Number of overlapping SNPs per panel after quality control.**

| **SNP panel** | **22k** | **26k** | **27k_1** | **27k_2** | **29k** | **30k** | **35k_1** | **35k_2** | **50k_1** | **50k_2** | **70k_1** | **70k_2** | **HD** |
| --- | --- | --- | --- | --- | --- | --- | --- | --- | --- | --- | --- | --- | --- |
| **14k** | 3,237 | 4,919 | 5,166 | 8,688 | 8,905 | 5,203 | 1,880 | 1,883 | 6,254 | 5,192 | 5,227 | 8,743 | 9,329 |
| **22k** |  | 3,512 | 3,960 | 3,447 | 3,752 | 4,037 | 2,746 | 2,751 | 11,848 | 5,530 | 4,498 | 11,637 | 15,179 |
| **26k** |  |  | 7,391 | 6,241 | 6,510 | 20,021 | 8,009 | 8,012 | 6,120 | 12,243 | 14,839 | 6,854 | 19,380 |
| **27k_1** |  |  |  | 5,450 | 5,778 | 7,722 | 6,633 | 6,644 | 5,507 | 10,817 | 11,109 | 6,197 | 22,318 |
| **27k_2** |  |  |  |  | 16,395 | 6,787 | 2,891 | 2,900 | 6,479 | 6,522 | 6,555 | 14,073 | 17,093 |
| **29k** |  |  |  |  |  | 7,164 | 2,878 | 2,890 | 6,912 | 6,653 | 6,781 | 15,445 | 18,282 |
| **30k** |  |  |  |  |  |  | 8,539 | 8,606 | 7,174 | 13,054 | 15,145 | 8,170 | 22,244 |
| **35k_1** |  |  |  |  |  |  |  | 31,319 | 2,452 | 31,304 | 11,954 | 4,001 | 29,882 |
| **35k_2** |  |  |  |  |  |  |  |  | 2,458 | 31,377 | 11,951 | 3,986 | 30,621 |
| **50k_1** |  |  |  |  |  |  |  |  |  | 7,292 | 7,263 | 22,799 | 26,244 |
| **50k_2** |  |  |  |  |  |  |  |  |  |  | 17,363 | 9,080 | 43,051 |
| **70k_1** |  |  |  |  |  |  |  |  |  |  |  | 9,251 | 62,955 |
| **70k_2** |  |  |  |  |  |  |  |  |  |  |  |  | 40,059 |

**Table S3. Number of overlapping SNPs between the 120k panel and commercial panels.**

| **SNP Panel** | **SNPs in common** |
| --- | --- |
| 14k | 9,329 |
| 22k | 15,179 |
| 26k | 19,380 |
| 27k_1 | 22,318 |
| 27k_2 | 17,093 |
| 29k | 18,282 |
| 30k | 22,244 |
| 35k_1 | 29,882 |
| 35k_2 | 30,621 |
| 50k_1 | 26,244 |
| 50k_2 | 43,051 |
| 70k_1 | 62,955 |
| 70k_2 | 40,059 |
| HD | 120,615 |

**Figure S3:** **Visual representation of the MAF distribution for the HD panel.**

**
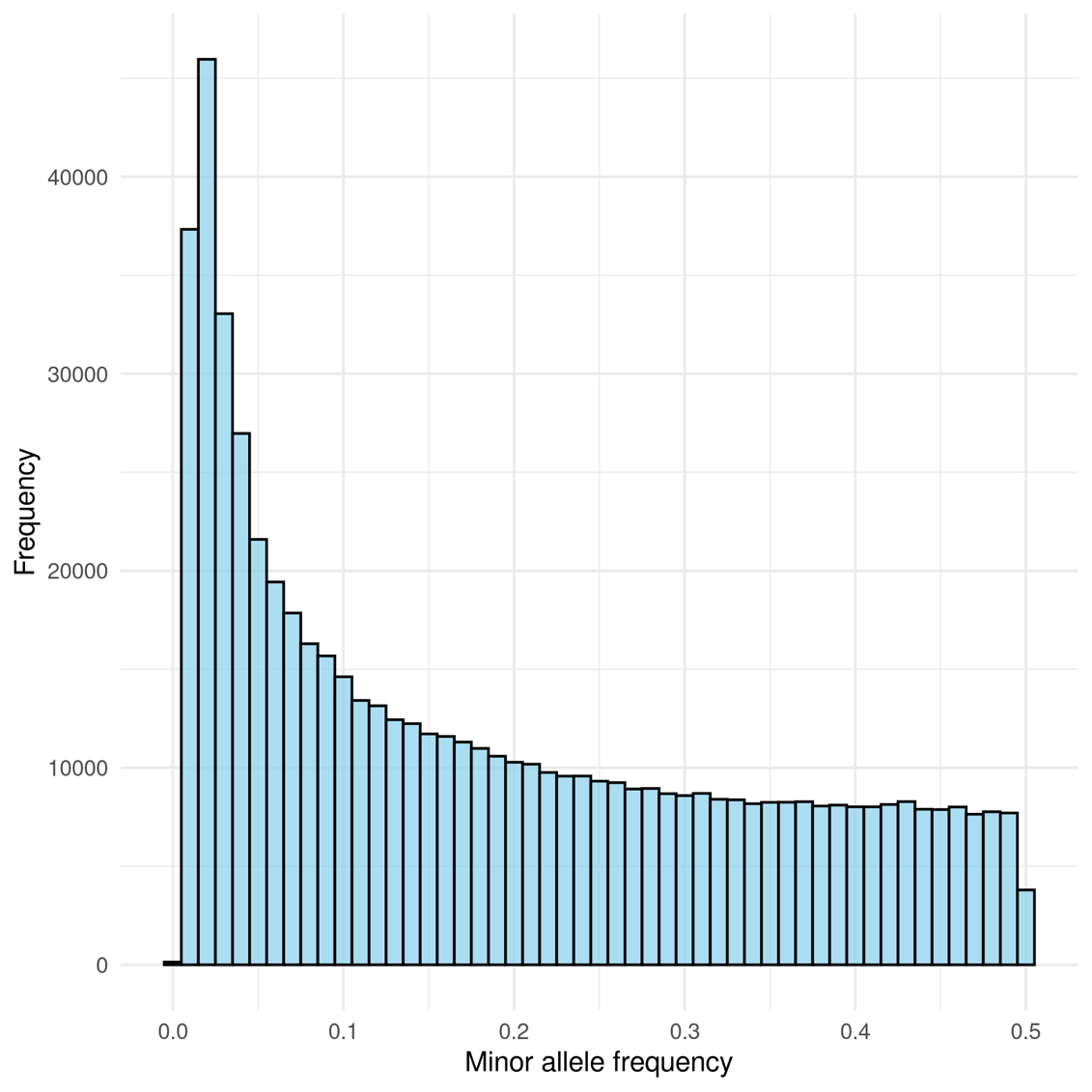
**
